# Impact of COVID-19 on the Participation of Iranian Adolescents in Daily Life: Development and Validation of the IAPAT-C Tool

**DOI:** 10.1155/2024/5583991

**Published:** 2024-10-16

**Authors:** Ghodsiyeh Joveini, Laleh Lajevardi, Mitra Khalafbeigi, Afsoon Hasani Mehraban, Armin Zareiyan

**Affiliations:** ^1^Department of Occupational Therapy, School of Rehabilitation Sciences, Kermanshah University of Medical Sciences, Kermanshah, Iran; ^2^Department of Occupational Therapy, School of Rehabilitation Sciences, Iran University of Medical Sciences, Tehran, Iran; ^3^Research Center for Cancer Screening and Epidemiology, Health in Disasters and Emergencies Department, School of Nursing, Aja University of Medical Sciences, Tehran, Iran

**Keywords:** adolescent, COVID-19, daily life, participation, reliability, validity

## Abstract

**Background and Purpose**: The present study is aimed at evaluating the effects of the COVID-19 pandemic on Iranian adolescents' participation in various domains of life. The electronic version of the Iranian Adolescent Participation Assessment Tool-COVID-19 (IAPAT-C) was developed and validated for this purpose.

**Methods**: This study was conducted in two phases. In the first phase, the electronic version of the IAPAT-C was developed and validated through a methodological study involving five stages: content validity review, cognitive interviews, item analysis, structural validity assessment using confirmatory factor analysis, and reliability analysis. The tool was adapted from the previously validated IAPAT and revalidated for this study. In the second phase, the effects of COVID-19 on adolescents' participation were evaluated using a longitudinal one group before and after comparative design. The study involved 654 adolescents aged 13–18, and the data were analyzed using SPSS19 and G⁣^∗^Power 3 software.

**Results**: The IAPAT-C evaluates the participation of Iranian adolescents in 54 daily life activities before and after the COVID-19 pandemic. It utilizes a 5-point Likert scale and was validated through an expert panel review for content validity. Additionally, cognitive interviews with six adolescents confirmed its comprehensibility, relevance, and comprehensiveness. Item analysis, based on data from 38 participants, demonstrated strong interitem correlations (0.6–0.94) and excellent internal consistency (Cronbach's alpha > 0.7). The tool's structural validity was supported by confirmatory factor analysis, which showed that the measurement model was a good fit. Convergent and discriminant validity of model constructs were also confirmed. Notably, COVID-19 significantly impacted all aspects of adolescents' lives, except for work.

**Conclusion**: The electronic version of the IAPAT-C was valid and reliable. COVID-19 significantly affected various aspects of Iranian adolescents' daily lives. Accordingly, it is necessary to provide appropriate interventions and policies for this sensitive class.

## 1. Introduction

The COVID-19 pandemic in 2020 posed significant challenges to the world. Home quarantine, social distancing, and a considerable focus on health issues have seriously affected individuals' personal and social lives, as the occupations of all people have been disrupted [1]. In this regard, it is crucial to consider vulnerable groups like adolescents who are experiencing the transition period and encountering the physical and emotional challenges of puberty [2]. Many researchers have studied the effects of the COVID-19 epidemic on personal and social lifestyles, as well as children and adolescents' mental and physical health [3–10]. Therefore, it is necessary and useful to investigate the effect of COVID-19 on different aspects of adolescents' daily occupations from an occupational therapy perspective to provide appropriate interventions and policies.

Home quarantine and school lockdown began on February 22, 2020, and continue to this day. These measures were taken to prevent the spread of infection in Iran. They have led to school lockdowns, closure of recreational and public places, long-term social distancing, and increased interaction between adolescents and their parents and families. In addition, the economic and work crises faced by parents have put stress on adolescents [11, 12]. These situations, along with the stress adolescents face due to the risk of getting sick or the illness of their family members and other influential people, have completely affected the experience of the next generation [2, 13].

The Iranian Adolescent Participation Assessment Tool (IAPAT) was initially developed to evaluate adolescents' participation in daily life activities. Specifically, it examines Iranian adolescents' engagement in 54 activities across various areas of occupation, aligning with the Occupational Therapy Practice Framework (OTPF-3). The tool underwent construct validity assessment through exploratory factor analysis, resulting in 54 items distributed across 7 domains. Notably, the internal consistency among Iranian adolescents was high (Cronbach's alpha = 0.92), and test–retest reliability ranged from 0.68 to 0.89 over a 1-week interval [14].

The IAPAT serves as a valuable instrument for studying the impact of events and stressors on adolescents' daily life participation. These stressors include natural disasters (e.g., floods and earthquakes), social threats (such as war or street clashes), psychosocial disorders (depression and psychosis), physical illnesses (cancer, heart disease, and stroke), and significant life changes (e.g., family dynamics and moving to a new house or school) [14, 15].

However, the unique circumstances of the COVID-19 pandemic necessitated the development of a new version of this tool, the Iranian Adolescent Participation Assessment Tool-COVID-19 (IAPAT-C). With home quarantine, school closures, and increased family interactions, face-to-face assessments became challenging. Thus, the IAPAT-C was designed—an electronic version—to assess Iranian adolescents' participation in daily life before and after the pandemic outbreak. The IAPAT-C retains the comprehensiveness and validity of its predecessor, allowing researchers to explore the pandemic's impact on adolescent participation.

Therefore, the objectives of this research were twofold as follows: (1) to design and validate the electronic version of the IAPAT-C and (2) to investigate the effect of COVID-19 on Iranian adolescents' participation in various aspects of life. This study is aimed at providing appropriate interventions and policies for this sensitive population in light of the significant changes brought about by the COVID-19 pandemic.

## 2. Method

The study was conducted in two main phases: First, the electronic version of the tool was designed and validated at five stages based on the methodological study, and second, the effects of COVID-19 on adolescents' participation were investigated based on the one-group before-after comparative design.

### 2.1. Phase 1: Developing and Validating the Electronic Version of the IAPAT-C in Daily Life

#### 2.1.1. Stage 1: Creating the Tool and Assessing Its Content Validity

The content validity of the tool was reviewed during its development in five steps at the suggestion of the COSMIN guideline [16] and based on the ISPOR guideline [17]. In the content validity phase, we involved four experts with PhDs in occupational therapy and one epidemiologist expert specializing in tool validation.

1. Creating the draft instrument: Given the impact of the COVID-19 pandemic on adolescents' daily lives and the challenges of face-to-face assessments due to home quarantine and travel restrictions, an electronic version of the IAPAT was designed through an expert panel. This tool was intended to assess Iranian adolescents' participation in daily life before and after the COVID-19 outbreak [14]. The items of the electronic version were adapted from the IAPAT and revalidated for use in this study.
2. Designing the cognitive interviews: The cognitive interviews were designed to evaluate the validity of the tool content from the adolescents' viewpoint through an expert panel. The questions focused on three main aspects of the tool content: comprehensibility, relevance, and comprehensiveness.

• Comprehensibility: This refers to whether the adolescents understood the items in the tool. Questions were designed to assess if the wording, language, and phrasing of the items were clear and easily understood by the adolescents.
• Relevance: This refers to whether the adolescents found the items in the tool to be relevant and applicable to their experiences during the COVID-19 pandemic. Questions were designed to assess if the items accurately reflected the adolescents' experiences and challenges.
• Comprehensiveness: This refers to whether the adolescents felt that the tool covered all the necessary areas of their daily life. Questions were designed to assess if any important aspects were missing from the tool.

The design of the cognitive interviews was a crucial step in ensuring that the tool was valid and accurately measured the impact of COVID-19 on Iranian adolescents' participation in various aspects of life.

3. Conducting a cognitive interview: After coordinating with the participants, a telephone call was made to each adolescent immediately after they completed the questionnaire. The interviewer and the participants reviewed all parts of the questionnaire according to the predesigned questions. Adolescents' comments and correction tips for each section were carefully noted by the interviewer.
4. Deciding to change the items: After conducting four interviews, the adolescents' opinions were reviewed through an expert panel. The sampling was stopped after six interviews, as there were no changes in the tool's items during the last two interviews.
5. Completing the item–tracking matrix: An item–tracking matrix was completed, which included the initial and final forms of the items, the changes made, and a quota sampling of the participants.

#### 2.1.2. Stage 2: Item Analysis

The purpose of item analysis is twofold as follows: to determine which items should be retained and to identify any need for additional items. Our overarching goal is to create a concise set of items for each construct dimension, ensuring acceptable internal consistency for the overall scale or its subscales [18]. During the pilot study, we meticulously analyzed the characteristics of each item in the IAPAT-C, following a three-step process:

1. Descriptive statistics assessment: We assessed descriptive statistics for each item, including response percentages, missing values, and measures of ceiling and floor effects. Desirable items exhibit means close to the center of the possible score range (e.g., a mean near 3.0 on a 5-point scale). Our aim was to achieve good variability on each item, ideally following a near-normal distribution of responses. Floor and ceiling effects were evaluated by scrutinizing extreme responses (highest or lowest). Items with a standard deviation less than 0.25 were flagged for potential deletion due to low variance. Items with more than 15% missing responses should be revised or replaced [19]. Average item correlation (AIC) values also should fall within the range of 0.2–0.4.
2. Correlational analysis: In our scale validation based on classical test theory (CTT), we sought to identify items highly correlated with the true score of the underlying construct. Interitem correlations were evaluated by examining the correlation matrix of all items or, if multidimensional, the items within hypothesized subscales. Correlations below 0.30 suggest limited congruence with the underlying construct, while those above 0.70 indicate redundancy [20].
3. Internal consistency assessment: We assessed the internal consistency of the IAPAT-C using two reliability indices: Cronbach's alpha and McDonald's omega. Higher values for these indices indicate greater reliability in capturing adolescents' participation during the COVID-19 pandemic [21].

#### 2.1.3. Stage 3: Structural Validity

In order to determine the structural validity and confirm the dimensions of the electronic version of the tool, we employed confirmatory factor analysis (CFA) using AMOS24 software. Given the comprehensiveness of the new tool, we utilized multiple fit indices. Distribution charts and Mahalanobis distance (*p* < 0.001) were used to identify both univariate and multivariate outliers. We also examined the normality of the data, considering skewness (values within ± 3), kurtosis (values within ± 7), and the Mardia coefficient (< 8). The study's data did not significantly deviate from a normal distribution. Additionally, we checked for missing data, but none was found since the tool was completed online [22].

#### 2.1.4. Stage 4: Reliability Analysis

After conducting the CFA and applying the necessary corrections, we aimed to determine the reliability of the electronic questionnaire. It was completed by 25 adolescents under similar conditions to ensure consistency. The conditions included a quiet environment, no time limit, and the same application and device used for the electronic questionnaire.

The test–retest evaluation method was employed, with an interval of 1 week between the two tests. This interval was chosen to minimize the possibility of the respondents remembering their previous answers, while also being short enough to ensure that no significant changes in their perceptions occurred [18, 20, 23, 24].

The intraclass correlation coefficient (ICC) was calculated to examine the stability, providing a measure of the test-retest reliability. This statistical technique assesses the consistency of different test results when the test is applied repeatedly.

In addition to the ICC, Cronbach's alpha was used to check the internal consistency of the questionnaire. This measure determines how closely related a set of items are as a group, providing an indication of the average correlation among all the items on the scale.

These methods allowed us to thoroughly evaluate the reliability of the electronic questionnaire, ensuring its suitability for further use in our study.

### 2.2. Phase 2: Investigation of the Effect of COVID-19 on Adolescent Participation

The second phase of the study was designed as a longitudinal one group before after comparative analysis to investigate the effect of COVID-19 on adolescent participation in daily life. The hypothesis tested was that adolescents' participation in daily life differed before and after the onset of COVID-19.

Data were analyzed using SPSS19, and the normality of the data was evaluated using two indices: skewness and kurtosis. The responses to daily activities before the COVID-19 pandemic were assessed retrospectively, meaning the adolescents were asked to recall their participation in daily activities prior to the pandemic.

To evaluate the difference in adolescent participation in each domain before and after the pandemic, a paired *t*-test was used. This statistical test compares the means of two related groups to determine if there is a significant difference between them.

Finally, G⁣^∗^Power 3 software was used to measure the effect size, which quantifies the size of the difference between groups. Effect size is a critical tool in reporting scientific results as it describes the magnitude of the effect independent of the sample size [25].

#### 2.2.1. Participants

The inclusion criteria were as follows:

A. Iranian adolescent girls and boys aged 13–18 in the seventh to twelfth grades,
B. Access to cyberspace, and the possibility of answering the electronic questionnaire.

After obtaining the relevant permits from the Tehran Education Department, six female and male first and second-grade high schools were randomly selected from the seventh to twelfth years (based on the Iranian educational system), where educational content was electronically provided, and school students had access to virtual educational channels. The electronic version of IAPAT in daily life was used in educational channels to assess the effect of COVID-19 in coordination with school officials. The adolescents were invited to log in to the link and complete the questionnaire. The questionnaire was completed by 654 adolescents, and the results were sent to the researcher for 1 week from April 18–25, 2020.

#### 2.2.2. Sampling Method and Sample Size Calculation

The study was conducted in two main phases, each with its own sampling method.

### 2.3. Phase 1: Developing and Validating the Electronic Version of the IAPAT-C in Daily Life

In the first phase, the sample consisted of adolescents who were selected based on the following inclusion criteria: (A) Iranian adolescent girls and boys aged 13–18 in the seventh to twelfth grades and (B) access to cyberspace and the possibility of answering the electronic questionnaire.

The sampling method in this phase was convenience sampling, where participants were selected based on their availability and willingness to participate. This method was chosen due to the practical constraints of the study, such as the need for participants to have access to cyberspace and the ability to answer the electronic questionnaire.

### 2.4. Phase 2: Investigation of the Effect of COVID-19 on Adolescent Participation

In the second phase, the sample size was calculated based on the results of the first phase and the statistical power needed for the longitudinal one group before and after the comparative design. The sample size calculation was based on the effect size (acceptable minimum), alpha level (probability of Type I error; 0.95), power (1—probability of Type II error; 0.95), and a sample size of 327 using G⁣^∗^Power 3 software. For the purpose of multivariate analysis, data from 654 participants were analyzed. The specific formula used for the sample size calculation and the values of the effect size, alpha level, and power used in the calculation can be provided upon request.

The sampling method in this phase was also convenience sampling, with the same inclusion criteria as in the first phase. The participants in this phase were not necessarily the same as those in the first phase.

## 3. Results

In the present study, 654 thirteen to eighteen years old Iranian adolescents participated, and Table 1 presents their demographic information.

### 3.1. The First Phase: Design and Validation of the E-Tool

#### 3.1.1. Stage 1: Design and Validation

##### 3.1.1.1. Step 1: Designing the Items

The Porsline Group (an Iranian team designing online questionnaires) was selected to provide a space to design and send the tool after consulting with artificial intelligence and computer software experts to investigate different methods of designing a web-based questionnaire. According to studies, there are limitations to electronic tools. For example, each item is presented to the audience on one page. Therefore, the following changes were made in designing the tool according to the research team to be adapted to the features of the electronic version.

In the IAPAT, the adolescents were asked to rate their participation in 54 activities. Changes were made to the items of the questionnaire to evaluate the effect of COVID-19 on adolescents' participation. Thus, in IAPAT-C, the adolescents were asked to rate their participation in each activity before and after COVID-19 (from *never* or *very low* to *very high*). Accordingly, two questions for each activity were asked about adolescents' participation before and after COVID-19. Therefore, the electronic version of IAPAT-C contained 54 items that were completed by adolescents before and after the COVID-19 pandemic (a total of 108 items) to investigate the effects of COVID-19 on adolescents' participation in daily life. Modifications and changes were made in the presentation of items in the electronic version of the tool.

##### 3.1.1.2. Step 2: Designing Cognitive Interviews

Some questions were designed in each section of the tool to examine the comprehensibility, relevance, and comprehensiveness of the items, along with their purpose.

##### 3.1.1.3. Step 3: Conducting Cognitive Interviews

A total of six cognitive interviews were conducted with adolescents who completed the questionnaire. This number falls within the range recommended by the COSMIN guideline for cognitive interviews. During these interviews, the adolescents' understanding of the items, the relevance of the items to their experiences, and the comprehensiveness of the tool were assessed. The feedback from these interviews was used to refine the tool. After six interviews, no new information was added (data saturation), and no changes were made to the tool's items during the last two interviews, indicating that the number of interviews was sufficient.

##### 3.1.1.4. Step 4: Deciding About the Tool Modification

Regarding the evaluation of the changes by experts, the adolescents' opinions were reviewed in an expert panel after four interviews. Based on the interviews, all sections of the questionnaire were comprehensible, relevant, and comprehensive from the adolescents' perspectives. Two participants shared a common view on a work-related activity to make it more comprehensible. This activity was studied and modified by researchers. The modified questionnaire was examined from different aspects during telephone interviews.

As for obtaining permission from the original owner to modify the items, the items of the electronic version were adapted from the IAPAT, which was designed by our research team. Therefore, there was no need to obtain permission from an external party to modify the items. We will add this information to the manuscript to clarify the process of item modification and the involvement of experts in this process.

##### 3.1.1.5. Step 5: Completing the Item–Tracking Matrix

Item–tracking matrix which shows the process of item modification was designed.

#### 3.1.2. Stage 2: Item Analysis

Using a pilot study, item analysis was examined before conducting structural validity. Our study had no missing values due to the online completion of the tool. We used the corrected item–total correlation approach in our study. All of the items in each seven subscales had an item–total correlation upper than 0.20. Internal consistency based on Cronbach's alpha and McDonald's omega, respectively, were 0.910 and 0.878. Another finding is shown in Table 2. All of the indexes are in the acceptable range; hence, no change was made to the tool at this stage [21].

#### 3.1.3. Stage 3: Structural Validity

Considering that the construct validity of the tool was previously determined through exploratory factor analysis, CFA was used in this study to confirm the structure of the tool [14]. After performing the CFA, six items whose standard factor loading was low were removed.

**Figure 1:**
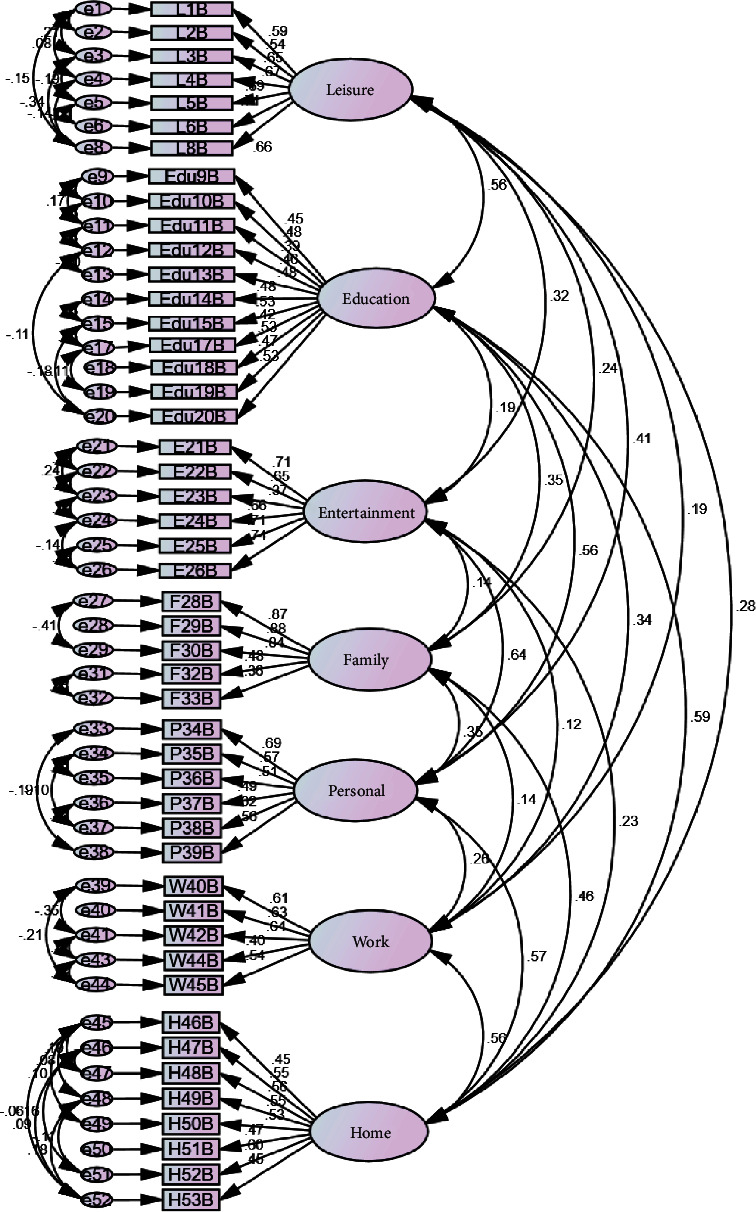
First order CFA (*n* = 654).

The goodness-of-fit indices of the model were evaluated. Myers et al. [26] consider it very important to report RMSEA, CFI, NFI, and chi-square values. Based on this, if at least three indicators are in the acceptable range, the fit of the model is acceptable. Thus, based on the results stated in Table 3, the structural validity of the tool was confirmed (Figure 1).

The reliability of the seven factors of this scale was excellent based on the composite reliability (CR). Based on the results of convergent validity, the AVE of seven factors was more than the MSV and shows that the factors have good convergent, but no discriminant validity except the familial activity domain.

#### 3.1.4. Stage 4: Reliability Evaluation

An electronic tool was completed twice by 25 adolescents 1 week apart to evaluate the reliability of the test–retest and internal consistency (Table 4).

### 3.2. Phase 2: Investigating the Effect of COVID-19 on Adolescents' Participation

**Figure 2:**
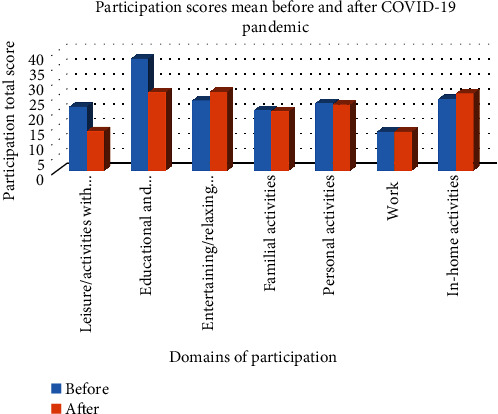
Comparison of the participants' scores before and after COVID-19.

In the second phase of the study, we investigated the effect of COVID-19 on adolescent participation in daily life using a longitudinal one group before and after comparative analysis. The hypothesis tested was that adolescents' participation in daily life differed before and after the onset of COVID-19. Table 4 presents the comparison of adolescent participation in each domain before and after the COVID-19 pandemic. The paired *t*-test results indicate that there was a significant difference in adolescent participation in all domains except for the post-COVID-19 work. This suggests that the COVID-19 pandemic had a significant impact on adolescent participation in daily life activities, with the exception of work-related activities. However, it is important to note that while the results were statistically significant, the effect size of COVID-19 on adolescent participation was weak to very weak in four domains (familial activities, personal activities, in-home activities, and work). This is also presented in Table 5. This suggests that while COVID-19 did have an impact on adolescent participation, the magnitude of this impact was relatively small in these domains. On the other hand, leisure/activities with friends, educational and school-related activities, and entertaining/relaxing activities were greatly affected. These findings provide valuable insights into the effects of the COVID-19 pandemic on adolescent participation in daily life. They highlight the need for further research and interventions to support adolescents during this challenging time. Our results showed that adolescent participation significantly changed in all domains except for the post-COVID-19 work. The mean total scores in each domain before and after the COVID-19 outbreak are presented in Figure 2. In conclusion, our findings suggest that the COVID-19 pandemic had a significant but relatively small impact on adolescent participation in daily life activities, highlighting the resilience of this population and the importance of continued support and interventions.

## 4. Discussion

The IAPAT-C plays a pivotal role in understanding how the COVID-19 pandemic impacts Iranian adolescents' participation across various life domains. Rigorous content validation ensures that the IAPAT-C captures essential aspects of adolescent participation. Experts meticulously reviewed and refined the items, ensuring their relevance and comprehensiveness. Adhering to the COSMIN methodology, we systematically evaluated the tool's quality during development, assessing construct relevance, clarity, and comprehensiveness. CFA confirmed the underlying structure of the IAPAT-C. Its dimensions align with our theoretical framework, supporting construct validity. Adequate factor loadings indicate effective measurement of intended constructs. Cronbach's alpha coefficients demonstrated satisfactory internal consistency, ensuring that items within each dimension consistently measured the same underlying construct. The IAPAT-C exhibited stability over time, crucial for assessing reliability. Adolescents' responses remained consistent when retested after an appropriate interval. Researchers and practitioners can confidently use the IAPAT-C to explore adolescent participation during the pandemic, given its robust psychometric properties.

The IAPAT-C benefits not only from robust methodology but also from its direct lineage to the IAPAT. The IAPAT was purposefully designed to evaluate the participation levels of Iranian adolescents across diverse activities. Its strong validity ensures accurate measurement in various contexts and populations, establishing it as a reliable tool for assessing adolescent participation. We recognized the need to assess the unique impacts of COVID-19 on adolescent participation. Hence, we developed the IAPAT-C, inheriting the robust psychometric properties of the original tool. By doing so, the IAPAT-C remains reliable and valid, even in the context of pandemic-related disruptions. In fact, the IAPAT-C's lineage and robust methodology position it as a valuable instrument for understanding adolescent participation during the pandemic.

The IAPAT-C's electronic format was a strategic response to pandemic challenges. Home quarantine measures limited face-to-face interactions, making electronic data collection essential. Adolescents completed the questionnaire swiftly, minimizing response time. Automatic data storage reduced manual entry errors, ensuring accurate results [29]. In summary, the IAPAT-C provides valuable insights into adolescent participation during the pandemic. Researchers can confidently utilize it to inform policies and interventions that support adolescents' well-being.

The present study was the first research on the effect of COVID-19 on Iranian adolescents' participation in various domains of life. It was indicated that the pandemic affected their participation in most domains of daily life. The investigation of adolescents' participation in various domains of daily life indicated that their participation in domains, including leisure/activities with friends, educational and school-related activities, familial activities, and personal activities decreased significantly. The leisure/activities with friends and educational activities mostly included activities outdoors, and achieving such results was to be expected. In the field of familial activities, although home quarantine is expected to increase adolescents' interaction with the family and participation in activities in this domain, going to parties, which has been considerably reduced due to social distancing and prevention of the spread of infection, is an important activity in this domain according to Iranian culture. In the field of personal activities, activities related to appearance and personal hygiene have slightly increased; however, the use of jewelry, attention to clothing coordination, and listening to friends have decreased. This is expected due to home quarantine.

Furthermore, Iranian adolescents' participation has increased in entertaining/relaxing and in-home activities. These results appear to be due to home quarantine, travel restrictions, reduced leisure activities with friends, and decreased participation in school-related activities.

Work-related activities remained relatively unaffected during the pandemic. Adolescents' participation in activities such as job search, the feeling of the need for work, and unpaid or low-paid work persisted. It is worth noting that the sample participants in our study were economically from the middle and upper classes, which likely influenced this finding. The economic impact of COVID-19 on families, especially in less affluent social classes, may yield different results regarding work-related participation.

The pandemic posed unique challenges for adolescents. Reduced physical activity, diminished social interactions, and increased vulnerability due to stress affected their well-being. Adolescents could not discharge emotions and feelings through physical activities, and their interactions with friends decreased, potentially impacting their cardiovascular health [2]. Increased interaction with family members, who were also dealing with illness and economic stress, made them more vulnerable [4, 5, 7]. While educational and school-related activities could mitigate some effects, the lack of prepared infrastructure for online education hindered many adolescents [1]. Policymakers should prioritize psychological support for families and ensure equitable access to online education systems.

In summary, our study successfully designed and validated the IAPAT-C, providing a valuable instrument for assessing adolescent participation during pandemic conditions. By adapting the existing IAPAT, we addressed the unique challenges posed by COVID-19 and contributed to the field.

### 4.1. Limitations

#### 4.1.1. Internet Access and Sampling

A challenge arose due to the inapplicability of a portion of the target group without Internet access. During the early stages of the COVID-19 outbreak, virtual education was unavailable in most schools. Many students from lower economic and social classes lacked the necessary facilities for online participation. Consequently, our research population primarily consisted of economically and socially affluent classes. To enhance generalizability, future studies should include participants from diverse social backgrounds, considering expanded access to virtual channels.

#### 4.1.2. Online Questionnaire Design

Online questionnaires have inherent limitations. For instance, presenting each question on a single page may impact adolescents' responses. We addressed this by including audio explanations and highlighting essential parts of each question. Despite the virtual nature of interviews, strong cognitive interviews ensured robust results during the design and validation of the IAPAT-C. Structural validity assessments confirmed that IAPAT-C effectively evaluated adolescents' participation before and after the COVID-19 outbreak. When designing online questionnaires, addressing these challenges and implementing appropriate solutions is crucial [29–33].

## Figures and Tables

**Table 1:** Demographic information of participants.

**Steps**	**Age mean ± SD**	**Gender**		**Number of participants**
		**Male (%)**	**Female (%)**	
Content validity	16 ± 1.9	33.3	66.7	6
Item analysis	15.1 ± 1.6	47.4	52.6	38
Structural validity and hypothesis testing	15.01 ± 1.4	51.2	48.8	654
Reliability	16.5 ± 1.3	90	10	25
Total number of participants				723

**Table 2:** Item analysis indices.

**Dimensions**	**α**	**ω**	**Item means**	**AIC**
Leisure/activities with friends	0.828	0.823	2.628	0.399
Educational and school-related activities	0.824	0.811	2.203	0.297
Entertaining/relaxing activities	0.804	0.789	3.222	0.371
Familial activities	0.787	0779	3.404	0.391
Personal activities	0.714	0.712	3.614	0.306
Work	0.754	0.693	2.259	0.338
In-home activities	0.667	0.594	2.611	0.201

Abbreviations: *α*, Cronbach's alpha; *ω*, McDonald's omega; AIC, average interitem correlation.

**Table 3:** Fit indices of the CFA model after structure modification (*n* = 654).

**Indices**	**χ** ^2^	**d** **f**	**p**_**value**_	**CMIN/DF**	**RMSEA**	**PCLOSE**	**PNFI**	**PCFI**	**IFI**	**CFI**
CFA model	2315.44	1016	< 0.0001	2.27	0.044	> 0.05	0.713	0.784	0.872	0.87

**Table 4:** Test–retest reliability and internal consistency of the items.

**Dimension**	**Number of items**	**Cronbach's alpha**	**ICC**	**95% confidence interval**	**p** ** value**
Leisure/activities with friends	16	0.83	0.85	0.92 (upper), 0.77 (lower)	< 0.001
Educational and school-related activities	24	0.81	0.89	0.94 (upper), 0.83 (lower)	< 0.001
Entertaining/relaxing activities	14	0.81	0.89	0.94 (upper), 0.82 (lower)	< 0.001
Familial activities	12	0.79	0.83	0.94 (upper), 0.82 (lower)	< 0.001
Personal activities	12	0.75	0.86	0.92 (upper), 0.78 (lower)	< 0.001
Work	12	0.83	0.68	0.82 (upper), 0.48 (lower)	< 0.001
In-home activities	18	0.74	0.76	0.87 (upper), 0.62 (lower)	< 0.001
Total	108	0.92	—	—	—

**Table 5:** Comparison of adolescent participation before and after COVID-19.

**Dimension participation**	**Mean difference**	**Standard deviation difference**	**95% CI**	**t**	**d** **f**	**p** ** value**	**Effect size**	
							**Cohen's ** **d**	**Interpretation** ^a^
Leisure/activities with friends	8.33	7.38	7.76–8.89	28.85	653	< 0.001	1.3	Very strong
Educational and school-related activities	11.37	9.48	10.65–12.1	30.66	653	< 0.001	1.2	Very strong
Entertaining/relaxing activities	−2.76	4.19	3.08–2.44	−16.86	653	< 0.001	−0.66	Moderate
Familial activities	0.45	2.89	0.23–0.67	4.01	653	< 0.001	0.16	Very weak
Personal activities	0.54	2.5	0.35–0.73	5.56	653	< 0.001	0.22	weak
Work	0.05	2.71	1.56–0.26	4.91	653	0.624⁣^∗^	0.02	Very weak
In-home activities	−1.76	4.11	2.07–1.44	−10.93	653	< 0.001	−0.43	weak

^a^The following rule of thumb (combining Cohen [27] and Sawilowsky [28]) was used to interpret the data. 0.00 < 0.20—*very wea*k (Sawilowsky), 0.20 < 0.50—*weak* (Cohen), 0.50 < 0.80—*moderate* (Cohen), 0.80 < 1.20—*strong* (Cohen), 1.20 < 2.00—*very strong* (Sawilowsky), 2 or more—*extremely strong* (Sawilowsky).

⁣^∗^*p* > 0.05 is not significant.

## Data Availability

The data that support the findings of this study are available on request from the corresponding author, Dr. Armin Zareiyan.

## References

[1] Hammell K. W. (2020). Making Choices from the Choices we have: The Contextual-Embeddedness of Occupational Choice. *Canadian Journal of Occupational Therapy*.

[2] Bruining H., Bartels M., Polderman T. J., Popma A. (2021). COVID-19 and child and adolescent psychiatry: an unexpected blessing for part of our population?. *European Child & Adolescent Psychiatry*.

[3] Cui Y., Li Y., Zheng Y. (2020). Mental health services for children in China during the COVID-19 pandemic: results of an expert-based national survey among child and adolescent psychiatric hospitals. *European Child & Adolescent Psychiatry*.

[4] Dvorsky M. R., Breaux R., Becker S. P. (2021). Finding ordinary magic in extraordinary times: child and adolescent resilience during the COVID-19 pandemic. *European child & Adolescent Psychiatry*.

[5] Franic T., Dodig-Curkovic K. (2020). COVID-19, child and adolescent mental health–Croatian (in) experience. *Irish Journal of Psychological Medicine*.

[6] Liu J. J., Bao Y., Huang X., Shi J., Lu L. (2020). Mental health considerations for children quarantined because of COVID-19. *The Lancet. Child & Adolescent Health*.

[7] O'Sullivan K., Clark S., McGrane A. (2021). A qualitative study of child and adolescent mental health during the COVID-19 pandemic in Ireland. *International Journal of Environmental Research and Public Health*.

[8] Xiang M., Zhang Z., Kuwahara K. (2020). Impact of COVID-19 pandemic on children and adolescents' lifestyle behavior larger than expected. *Progress in Cardiovascular Diseases*.

[9] Xie X., Xue Q., Zhou Y. (2020). Mental health status among children in home confinement during the coronavirus disease 2019 outbreak in Hubei Province, China. *JAMA Pediatrics*.

[10] Zhou S. J., Zhang L. G., Wang L. L. (2020). Prevalence and socio-demographic correlates of psychological health problems in Chinese adolescents during the outbreak of COVID-19. *European Child & Adolescent Psychiatry*.

[11] Heidari M. (2020). The necessity of knowledge management in novel coronavirus (COVID-19) crisis. *Depiction of Health*.

[12] Mansori M., Pakar E., Karimizadeh Ardakani M., Mohammadkhani K. (2022). The effect of regular physical activity on aggression and quality of life of students during corona quarantine (COVID-19). *Iranian Journal of Health Education & Health Promotion*.

[13] Guessoum S. B., Lachal J., Radjack R. (2020). Adolescent psychiatric disorders during the COVID-19 pandemic and lockdown. *Psychiatry Research*.

[14] Joveini G., Hasani Mehraban A., Zareiyan A., Khalafbeigi M., Lajevardi L. (2022). Development and validation of Iranian adolescent’s participation assessment tool. *British Journal of Occupational Therapy*.

[15] Yeaworth R. C., McNamee M. J., Pozehl B. (1992). The adolescent life change event scale: its development and use. *Adolescence*.

[16] Terwee C. B., Prinsen C. A. C., Chiarotto A. (2018). COSMIN methodology for evaluating the content validity of patient-reported outcome measures: a Delphi study. *Quality of Life Research*.

[17] Patrick D. L., Burke L. B., Gwaltney C. J. (2011). Content validity--establishing and reporting the evidence in newly developed patient-reported outcomes (PRO) instruments for medical product evaluation: ISPOR PRO good research practices task force report: part 1--eliciting concepts for a new PRO instrument. *Value in Health: The journal of the International Society for Pharmacoeconomics and Outcomes Research*.

[18] Polit D. F., Yang F. M. (2016). *Measurement and the measurement of change*.

[19] DeVet H. C. W., Terwee C., Mokkink L. B., Knol D. L. (2011). *Measurement in medicine: A practical guide*.

[20] Streiner D. L., Norman G. R., Cairney J. (2015). *Health measurement scales: a practical guide to their development and use*.

[21] Salaree M. M., Zareiyan A., Ebadi A. (2019). Development and psychometric properties of the military nurses' job burnout factors questionnaire. *Journal of Military Medicine*.

[22] Hair J. F., Black W. C., Babin B. J., Anderson R. E. (2019). *Multivariate data analysis*.

[23] Deyo R. A., Diehr P., Patrick D. L. (1991). Reproducibility and responsiveness of health status measures: statistics and strategies for evaluation. *Controlled Clinical Trials*.

[24] Nunnally J. C., Bernstein I. H. (1994). *Psychometric theory*.

[25] Faul F., Erdfelder E., Lang A.-G., Buchner A. (2007). G^∗^Power 3: a flexible statistical power analysis program for the social, behavioral, and biomedical sciences. *Behavior Research Methods*.

[26] Myers N. D., Wolfe E. W., Feltz D. L. (2005). An evaluation of the psychometric properties of the Coaching Efficacy Scale for American coaches. *Measurement in Physical Education and Exercise Science*.

[27] Cohen J. (1988). *Statistical power analysis for the behavioral sciences*.

[28] Sawilowsky S. S. (2009). New effect size rules of thumb. *Journal of Modern Applied Statistical Methods*.

[29] Mudavath A., Narayan K. A. (2019). Strengths and Weakness of Online Surveys. *IOSR Journal of Humanities and Social Science(IOSR-JHSS)*.

[30] Andrade C. (2020). The limitations of online surveys. *Indian Journal of Psychological Medicine*.

[31] Shannon D. M., Johnson T. E., Searcy S., Lott A. (2002). Using electronic surveys: advice from survey professionals. *Practical Assessment, Research, and Evaluation*.

[32] Sincero S. M. (2012). Online surveys. https://explorable.com/online-surveys.

[33] Wright K. B. (2005). Researching Internet-based populations: advantages and disadvantages of online survey research, online questionnaire authoring software packages, and web survey services. *Journal of Computer-Mediated Communication*.

